# Recombinant human erythropoietin stimulates melanoma tumor growth through activation of initiation factor eIF4E

**DOI:** 10.18632/oncotarget.16331

**Published:** 2017-03-17

**Authors:** Annabelle Julius, Anjali Desai, Raymond L. Yung

**Affiliations:** ^1^ Division of Geriatric and Palliative Medicine, Department of Internal Medicine, University of Michigan, Ann Arbor, MI, USA; ^2^ Geriatric Research, Education and Clinical Center, Veterans Affairs Ann Arbor Health System, Ann Arbor, MI, USA

**Keywords:** melanoma, erythropoietin

## Abstract

Recombinant human erythropoietin (EPO) is standard treatment for anemia in cancer patients. Recent clinical trials suggest that EPO may accelerate tumor progression and increase mortality. However, the evidence supporting a growth-promoting effect of EPO has remained controversial. Employing an *in vivo* model of B16 murine melanoma, we observed that administration of EPO to tumor bearing C57BL/6 mice resulted in pronounced acceleration of melanoma growth. Our *in vitro* studies demonstrate that B16 murine melanoma cells express EPOR, both at the protein and mRNA levels. Interestingly, expression of EPOR was retained in the established tumors. EPO stimulation of B16 cells enhanced proliferation and protein synthesis rates, and correlated with activation of the receptor associated Janus kinase 2 (Jak2) as well as phosphorylation of extracellular signal–regulated kinase (Erk) 1/2 and Akt kinases. Treatment with EPO and Jak-2 antagonists significantly inhibited EPO-mediated B16 cell proliferation. Moreover, EPO dose-dependently induced the phosphorylation and activation of the translation initiation factor eIF4E as well as the phosphorylation of its repressor, the eIF4E binding protein 4E-BP1. Finally, using eIF4E small interfering RNA (siRNA), we observed that EPO-mediated stimulation of B16 cell proliferation is eIF4E-dependent. Our results indicate that EPO exerts a powerful stimulatory effect on cell proliferation and *de novo* protein synthesis in melanoma cells through activation of the initiation factor eIF4E.

## INTRODUCTION

Recombinant human erythropoietin is a glycoprotein hormone that serves as the primary regulator of red blood cell production by stimulating growth, preventing apoptosis, and inducing differentiation of erythroid progenitor cells. Since EPO was first purified from urine more than three decades ago, its recombinant form has become widely used for the treatment of anemia associated with chronic kidney disease, human immunodeficiency acquired immune deficiency syndrome (HIV/AIDS), and cancer. While treatment with EPO has significantly reduced the need for transfusions and has improved the overall quality of life for many cancer patients, reports from several recent clinical trials suggest that therapeutic doses of EPO or related erythropoiesis-stimulating agents (ESAs) may promote later tumor metastasis and mortality [1]. However, due to the diversity in trial design and variability in the patients’ disease stage and control groups, these reports have stirred great controversy among oncologists and there is an urgent need to further evaluate the clinical application of ESAs in cancer [2, 3]. There is accumulating evidence that the adverse effects of EPO may be a consequence of the ubiquitous expression of functional EPORs in cancer cells. Kumar et al. found that EPOR mRNA was expressed in more than 90% of 65 melanoma cell lines they tested [4]. Mirmohammadsadegh et al. demonstrated that EPOR is expressed in human melanoma specimens and expression is significantly higher is melanoma metastasis compared to nevi and primary melanoma suggesting an association with disease progression and EPOR expression [5].

Based on the current literature, the influence of EPO/EPOR on different cancer types appears to be variable and remains incompletely understood and more functional studies are needed to determine whether EPOR activation modifies tumor cell growth.

In erythroid cells, binding of EPO to its preformed homodimer receptor induces subsequent activation of a series of signaling molecules, such as Jak2 and signal transducer and activator of transcription 5 (Stat5) leading to increased proliferation and differentiation [6, 7]. The cellular mechanism of the action of EPO in cancer cells in contrast, appears not only potentially different from the one described in hematopoietic cells but also seems to vary among cancer cell types and even within cell types. For instance, although activation of Jak2 and/or Stat 5 was observed in head-neck cancer, neuroblastoma, prostate cancer (reviewed in [1]) and in a rat mammary cell line transfected with human EPOR [8], phosphorylation of Stat5 was not observed in MCF-7 and MDA-MB231 human breast cancer cell [9]. On the other hand, Zhou at al. very recently reported that EPO promoted tumorigenesis by activating JAK/STAT signaling in breast tumor-initiating cells and that high levels of endogenous EPO gene expression correlated with shortened relapse-free survival in a mouse model of breast cancer [10]. In addition, it has been reported that EPO induces phosphorylation of mitogen-activated protein kinase family members such as Erk-1/2 in human ovarian cancer cells [11] and phosphorylation of Akt in neuroblastoma cells [12] and breast cancer [8]. Although increased phosphorylation and thus activation of signaling kinases has been found in tumor cells in response to EPO treatment, a consensus has yet to be reached regarding its role in tumor proliferation (for a recent review see [13]).

Protein synthesis is an important determinant of cell proliferation and differentiation, and is regulated by hormones, growth factors, cytokines and mitogens through phosphorylation of different translation factors. With cellular levels 10- to 30-fold lower than those of other known initiation factors, eukaryotic initiation factor 4E (eIF4E) is considered to be the rate-limiting protein in translation initiation [14]. As eIF4E binds the cap structure present at the 5”end of mRNAs, it delivers these mRNAs to the eIF4F complex. This complex comprised of eIF4E, the ATP-dependent RNA helicase, eIF4A, and the scaffolding protein eIF4G then scans through and unwinds the 5′untranslated region of the mRNA to reveal the initiation codon and enable translation. Assembly of the eIF4F complex is dependent upon eIF4E availability and is regulated by eIF4E-binding proteins (4E-BPs) such as 4E-BP1 [15]. The 4E-BPs act as molecular mimics of the eIF4E-binding site in eIF4G, and thus effectively compete with eIF4G for eIF4E binding [16]. Binding of the 4E-BPs to eIF4E is reversible. Whereas hypophosphorylated 4E-BPs bind avidly to eIF4E, 4E-BP hyperphosphorylation by hormones and growth factors through the mammalian target of rapamycin (mTOR)/Akt pathway abrogates this interaction, allowing eIF4E to form the active eIF4F complex [17]. Activity of eIF4F is also regulated by phosphorylation of eIF4E [15] and several studies have documented that both eIF4F complex assembly and the subsequent phosphorylation of eIF4E play a crucial role in neoplastic transformed cells but not normal cells (reviewed by [18], [14, 19]). Interestingly, Bu et al. [20] reported that EPO stimulates eIF4E phosphorylation in erythroblasts, and phosphorylation of 4E-BP1 has recently been correlated with advanced pathologic grade and worse prognosis in human melanoma [21]. However, existence and relevance of these regulatory phosphorylation events in EPO-mediated signaling pathways leading to tumor progression has not been explored. The aims of the current study were to examine the effect of EPO on growth and protein synthesis rates in tumor cells and to elucidate the signal transduction pathways involved in these processes. To our knowledge, the present study is the first to demonstrate accelerated tumor growth in EPO-treated mice with melanoma. Our *in vitro* studies suggest that EPO-mediated activation of eIF4E may play an important role in tumor cell proliferation.

## RESULTS

### Administration of EPO increases melanoma tumor cell growth *in vivo*

**Figure 1:**
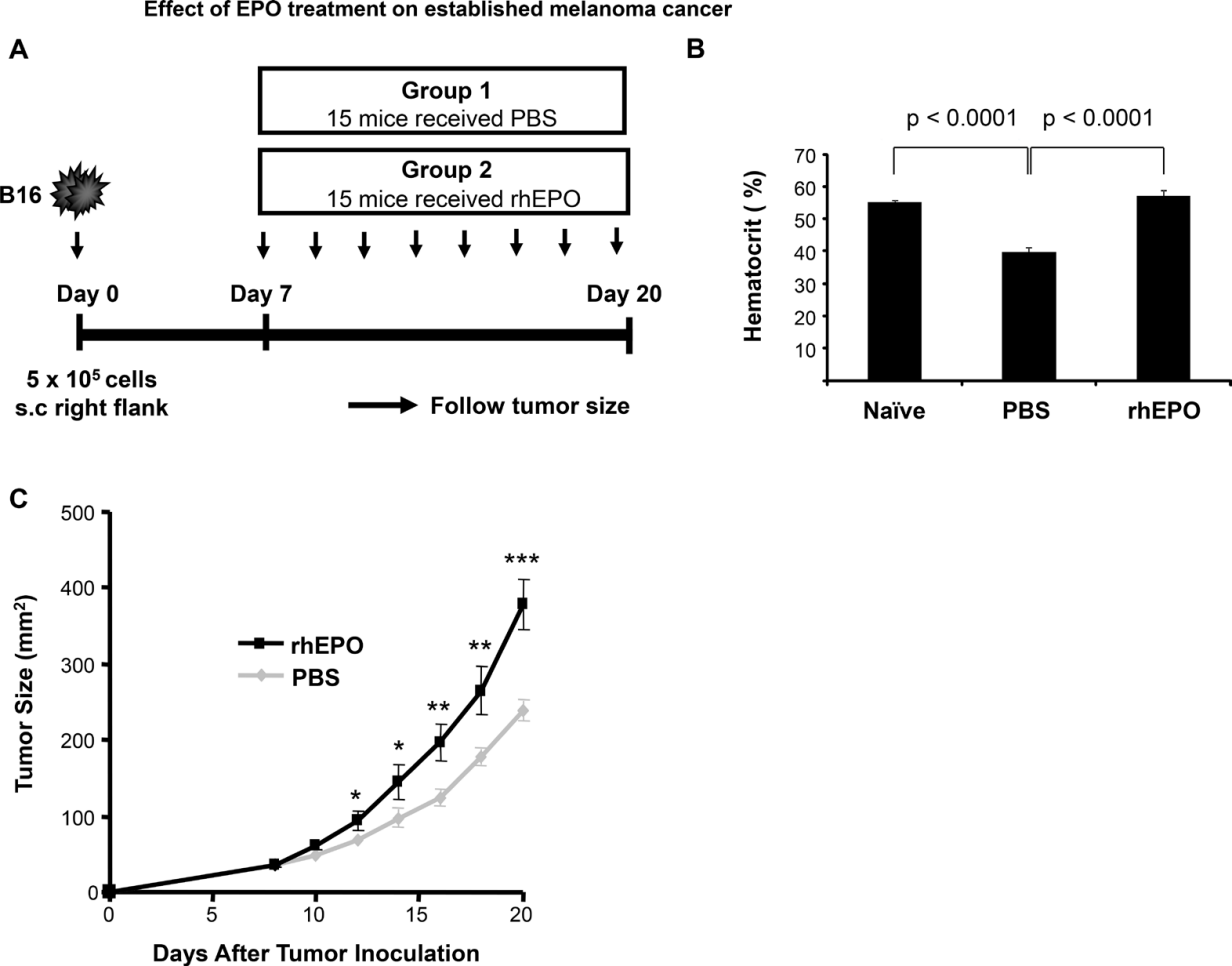
Effect of EPO treatment on established melanoma cancer (**A**) Model depicting experimental design (**B**) Comparison of hematocrit levels in rhEPO- and PBS-treated mice versus naïve mice. Hematocrits were measured as described in Materials and Method before tumor cells injection (naïve measurement) and at the endpoint of the experiment in the PBS- and EPO-treated mice. Bars represent mean levels of each group ± SEM from two independent experiments. (**C**) Data displayed is the mean of the tumor size measured at the indicated time point for all the mice and is representative of two independent experiments. * *p* < 0.05; and ** *p* < 0.005; *** *p* < 0.0005. *n* = 15 per group.

To test the effect of EPO on melanoma tumor growth *in vivo*, we used a well characterized B16 melanoma tumor model [22]. C57BL/6 mice were inoculated with B16 cells subcutaneously on day 0 and injected with PBS (control group) or EPO (30 U) [23] three times per week starting when the tumors were palpable. Tumor growth kinetics was then assessed (Figure 1A). We first confirmed erythropoiesis stimulating activity of EPO at the selected dose by measuring the hematocrit level in mice before and after 3 weeks of treatment. Baseline hematocrit of mice was 55.22 ± 0.69%. Following tumor cell inoculation and subcutaneous administration of PBS, a 28% decrease in hematocrit levels was observed by the end of the study period (Figure 1B). As expected, treatment of tumor-bearing mice with EPO resulted in a marked increase in hematocrit by the end of the study period when compared to PBS-injected control animals (56.9 ± 1.9% vs. 39.8 ± 1.38% ; Figure 1B) and to a level comparable to that of naïve mice. Figure 1C shows the results of melanoma tumor growth between the group receiving EPO and the control group. We found that EPO therapy significantly accelerated the development of established tumors in C57BL/6 mice as early as 12 days after tumor inoculation and thereafter compared to PBS-treated control mice. At day 20, the mean tumor size in the group receiving EPO was 37% greater than that in control mice (378 ± 33.9 vs. 238.5 ± 12.1; *p* < 0.0005).

### B16 melanoma cells express EPOR

**Figure 2:**
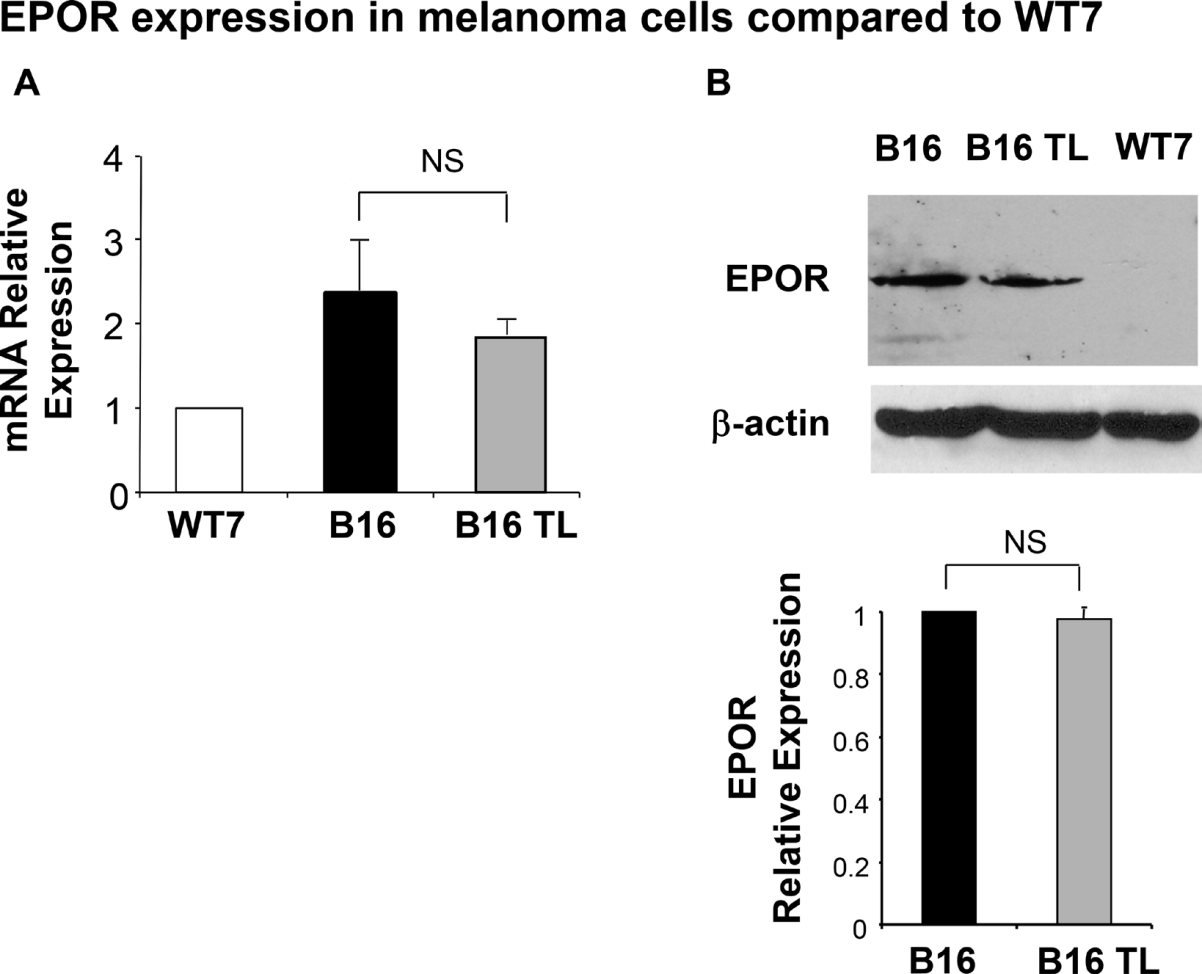
Expression of EPOR in B16 cells **(A)** EPOR transcripts from WT7 cells, B16 cells, and B16 tumor lysates from tumor-bearing mice (B16TL) were quantitated relative to GAPDH by real-time RT-PCR as indicated in Materials and Methods. Results represent the mean ± SEM of three different experiments, normalized to WT7 control. **(B)** A representative Western blot analysis is shown. EPOR expression was observed using an anti-EPOR antibody. WT7 lysate was run as an EPOR-negative control. The same blot was probed with an anti-β-actin antibody for loading control. Data are normalized to β-actin expression and represent the mean ± SEM of at least two samples per assay compared to cultured B16 cells whose values were normalized to 1.

We next examined the expression of EPOR in B16 cells. Using a murine keratinocyte cell line (WT7) as a negative control we observed that cultured B16 melanoma cells express EPOR at both the mRNA and protein level (Figure 2A and 2B). Importantly, EPOR expression levels both at the mRNA (Figure 2A, grey bar) and protein (Figure 2B, lane 2) levels remain unchanged in B16 tumors established in syngeneic C57BL/6 mice for 3 weeks (B16 TL).

### Erythropoietin induces B16 cell proliferation

**Figure 3:**
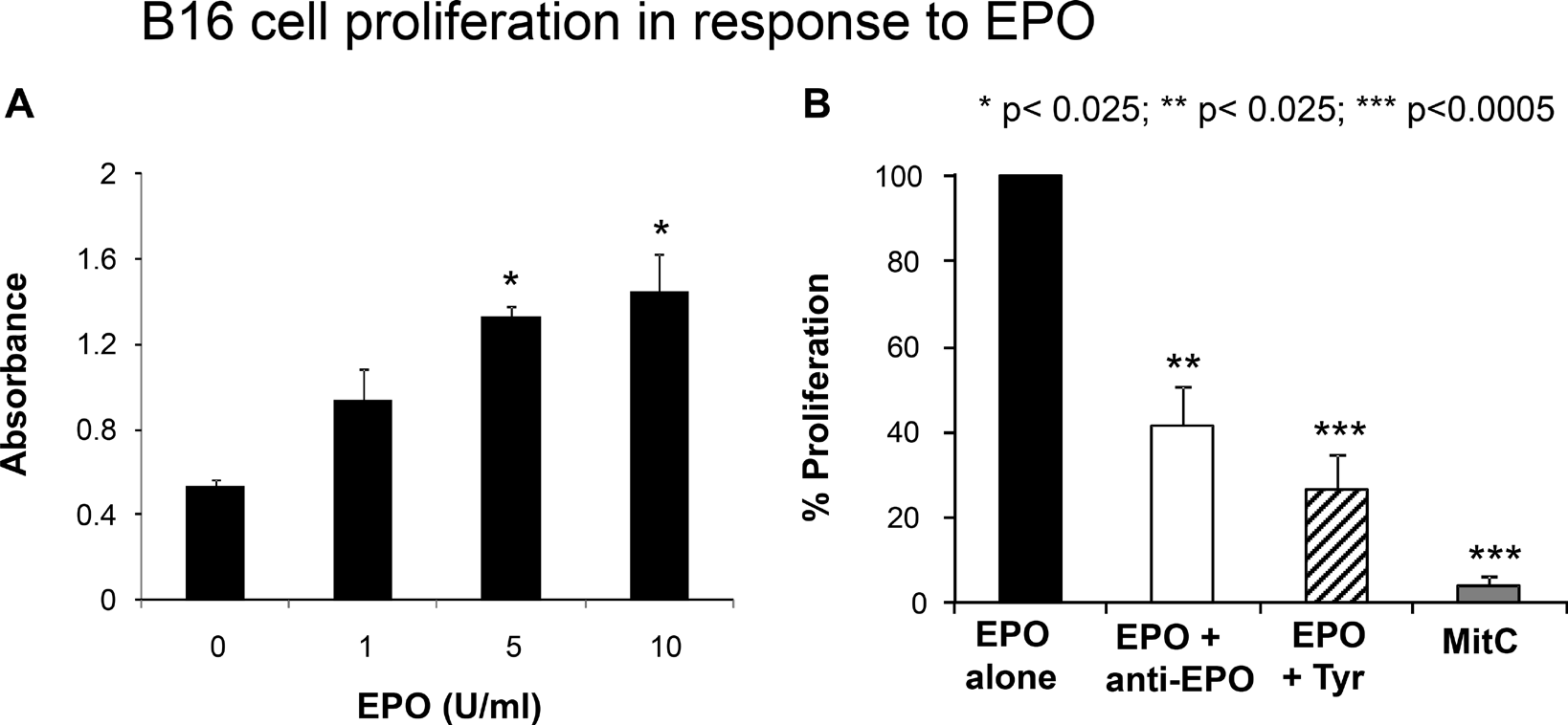
Functional studies of EPOR **(A)** B16 cells were incubated for 4 hours with increasing concentrations of EPO followed by BrdU administration.. Cell incorporation of BrdU was measured 12 hours later as described in Materials and Methods. Results of a representative experiment are presented as mean absorbance of quadruplicate determinations. Asterisks indicate significant difference compared to the untreated values. **(B)** B16 cells were treated with EPO (10 U/ml) after 1hour pretreatment without or with an anti-human EPO neutralizing antibody, tyrphostin AG 490 or the proliferation inhibitor Mitomycin C (MitC) as indicated. Proliferation was determined after 16 hours and results expressed as relative to the absorbance value of the EPO alone treatment (arbitrarily defined as equal to 100%). *p < 0.025; **p < 0.0025; ***p < 0.0005.

Several preclinical studies have reported direct effects of EPO on normally proliferating and on cancer cells such as activation of intracellular signal transduction or stimulation of proliferation, whereas other studies have found no significant effects [24–28]. To determine whether the EPOR expressed by B16 cells is functional in response to EPO treatment, we first examined cell proliferation in response to EPO by measuring BrdU incorporation. As shown in Figure 3A, increasing concentration of EPO from 1 to 10 U/ml induced a robust proliferative response in a B 16 cells dose-dependent manner. Maximal effect on cell proliferation was observed at 10 U/ml EPO. A 15 or 20 U/ml concentration did not have any added effect over the 10 U/ml concentration (data not shown). Importantly, treatment of B16 cells with a EPO-neutralizing monoclonal antibody prior to stimulation with EPO, significantly decreased cell proliferation by 57% compared to EPO-stimulated cells (Figure 3B). Similarly, pretreatment of the cells with tyrphostin, an inhibitor of Janus kinase 2 (JAK2), a tyrosine kinase that has been shown to be an essential part for most but not for all of the known receptor functions of EPOR [29, 30], decreased EPO-stimulated BrdU incorporation by 74% (Figure 3B). These results suggest that EPO-induced proliferation of B16 cells is mediated by an EPO-EPOR signaling pathway which requires activation of Jak2.

### Erythropoietin induces de novo protein synthesis

**Figure 4:**
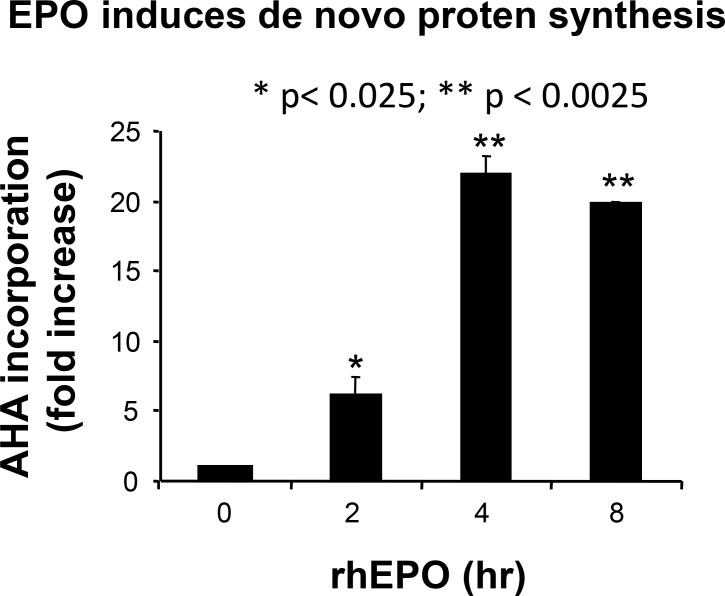
Effect of EPO on de novo protein synthesis B16 cells were treated with or without EPO (10 U/ml) for 2, 4 and 8 hours, respectively, in L-methionine-free medium containing 50 μM AHA. Cells were washed, fixed, permeabilized, and nascent protein synthesis measured by quantification of AHA incorporation following a click reaction with an Alexa fluor 488 alkyne. Results are expressed as fold increase (mean ± SEM) in incorporation compared to untreated cells (time 0) whose values were normalized to 1. *p < 0.005 and **p < 0.0005 vs. untreated cells.

As we and others have shown that protein synthesis rate is a major determinant of cell proliferation [31], we next analyzed the effect of EPO on protein synthesis rates. To detect nascent proteins, we used the nonradioactive reagent L-azidohomoalanine (AHA), a methionine analog that is incorporated into proteins instead of methionine and is detected by reaction of the azido-modified protein with a fluorescent alkyne (for details, see Materials and Methods). Incorporation of AHA into B16 cells treated with 10 U/ml EPO (the concentration that leads to the maximal effect of EPO in Figure 3A) was determined after 2, 4 and 8 hours of culture, respectively. As illustrated in Figure 4, protein synthesis in B16 cells was rapidly and strongly induced by EPO, with a 21.9 ± 1.3 and 19.8 ± 0.1 fold increase observed after 4 and 8 h of EPO treatment, respectively.

### EPO-EPOR signaling

**Figure 5:**
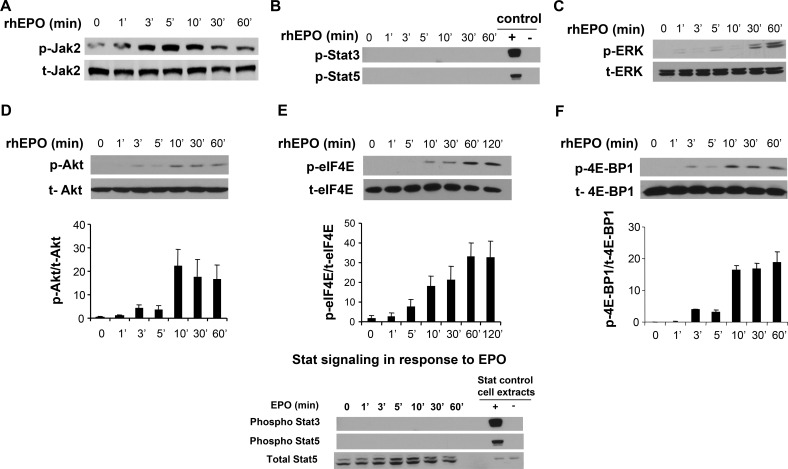
EPO signaling in B16 cells B16 cells were serum starved for 24 hours, stimulated with EPO (10U/ml) for the indicated period, and lysates subjected to SDS-electrophoresis and immunoblot analysis as described in Materials and Methods. Phosphorylation and total forms of Jak2 **(A)**, Stat3 and Stat5 **(B)**, Erk1/2 **(C)**, Akt **(D)**, eIF4E **(E)**, and 4E-BP1 **(F)** was then similarly compared by immunoblotting using specific antibodies directed against phosphorylated forms of the enzymes. A representative Western Blot of three independent experiments is shown. For the Western blot using anti-phospho Stat antibodies, cell lysates derived from serum-starved HeLa cells stimulated with IFN-α were used as positive controls, whereas HeLa cell extracts prepared without treatment served as negative controls. For panels (D), (E) and (F) immunoreactivity was quantified by scanning densitometry, and the ratio phospho/total was represented. Statistical significant differences compared to the untreated values were obtained starting at 3 minutes of treatment with EPO for p-Akt and p-4E-BP1, and at 10 minutes for p-eIF4E.

To further elucidate the molecular mechanism of EPO-mediated B16 cell proliferation, the activation status of the Jak2, Erk, and Akt kinases was investigated. Cells were stimulated with 10 U/ml EPO and time-dependent kinase activation was examined by Western blotting. Results of these experiments are shown in Figure 5. As expected based on the literature and the results of our proliferation experiment using a Jak2 inhibitor, EPO induced phosphorylation of Jak2 within minutes of treatment and without affecting its total expression level (panel A). Phosphorylation of Stat5 and Stat3, which has been reported in various tumor cells following EPO-mediated Jak2 activation [5] was next examined. As shown in panel B, although phosphorylation of Stat3 and Stat5 was observed in our positive controls, we did not observe any activation in response to EPO stimulation. In contrast, treatment of B16 cells with EPO led to phosphorylation of ERK1/2 (panel C) and Akt (panel D), with a more rapid induction of the phosphorylated state of Akt compared to ERK1/2. For Akt, phosphorylation was observed as early as 10 min after rhEPO stimulation. Total protein level was not affected by rhEPO treatment during the tested time period.

Interestingly, activation of Erk1/2 and Akt can lead to hyperphosphorylation of 4E-BP1, a repressor of eIF4E that plays a critical role in the control of protein synthesis and cell grow [32, 33]. Since both protein synthesis and proliferation rates were induced by EPO, we investigated the phosphorylation status of 4E-BP1 in EPO-stimulated B16. As illustrated in panel E, 4E-BP1 phosphorylation was detected after 3 minutes of exposure and kept increasing at later time points. More importantly, EPO also triggered the phosphorylation of eIF4E (panel F), an event that only can occur once eIF4E has been released by phosphorylated 4E-BP1 and that has oncogenic potential [34, 35]. This effect was maximal after 60 minutes of EPO stimulation and stayed well above basal level even after 120 minutes of stimulation.

### Erythropoietin-mediated stimulation of cell proliferation is eIF4E-dependent

**Figure 6:**
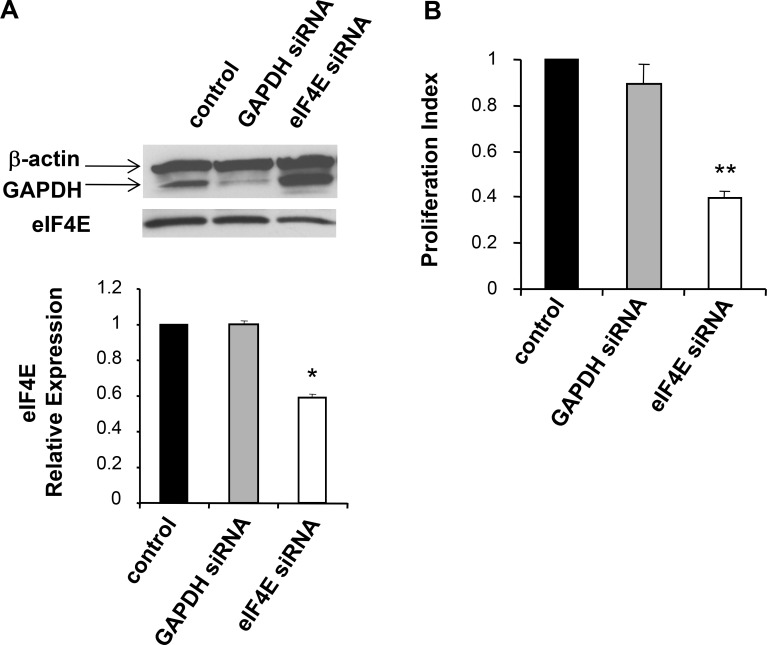
siRNA mediated knockdown of eIF4E in B16 cells **(A)** Three days post transfection with the indicated siRNAs, B16 whole cell lysates were prepared and knockdown of eIF4E assayed via western blot analysis. Equal loading of each lane was determined using β-actin expression. The histogram illustrates eIF4E relative expression in mock-transfected cells (control; arbitrarily define as equal to 1.0) or indicated siRNA-treated cells. (**B**) The effect of eIF4E siRNA on cell proliferation was assessed by BrdU assay. Values, reported as the percentage of BrdU incorporation in in EPO-treated cells transfected with specific eIF4E siRNA or non target siRNA (NT) relative to mock-transfected cells (control cells; arbitrarily define as equal to 100), are the means ± SEM of three separate experiments.

To determine the importance of eIF4E activation to the EPO-mediated proliferative effect, we used small interfering RNA (siRNA)-mediated knockdown of eIF4E. A siRNA against murine eIF4E or a control non-targeting (NT) siRNA respectively, were transfected into B16 cells. In order to evaluate the downregulation of eIF4E protein expression, Western blot analyses were performed three days later. As illustrated in Figure 6A, transfection of the eIF4E siRNA resulted in a decrease in eIF4E protein levels relative to the mock-transfected and to the NT-siRNA–transfected cells. No effects of siRNA were observed on the expression of β-actin used as an internal control. Next, we investigated the effect eIF4E siRNA on B16 cell proliferation. Three days post-transfection, cells were treated with EPO (10 U/ml) and cellular proliferation was evaluated through the incorporation of BrdU 12 hrs later. Figure 6B shows that, compared with control cells, % reduction in BrdU incorporation was detected after transfection of eIF4E siRNA. No obvious inhibition of proliferation was detected when cells were transfected with the NT-siRNA. These data confirm that EPO influences cell growth via eIF4E activation.

## DISCUSSION

In the present study we sought to explore the effect of EPO administration on the development of established murine melanoma. In order to validate the usefulness of the well-characterized B16 murine melanoma tumor model that we had previously established in our laboratory, we first confirmed expression of EPOR by two independent methods. Using both Western blotting and real time PCR, we observed that EPOR was not only expressed in B16 cells *in vitro* but that expression was also retained in established tumors *in vivo*.

Contrary to the recent observations by Kumar et al [4], we observed that EPO treatment also significantly accelerated melanoma growth *in vivo*. The EPO dose chosen for our experiments was based on previously published work from our laboratory and was comparable to that used by Kumar et al. The main differences between Kumar's and our study are the animal model and the dosing schedule that were used. While we induced B16 melanoma in wild-type mice, Kumar et al. injected EPOR-expressing 1232Lu melanoma cells into athymic nude mice [4]. Furthermore, Kumar et al. administered 2000 U/kg EPO on a daily basis whereas we injected mice subcutaneously with 30 U of EPO (between 1200 and 1500 U/kg) every other day [4].

In our studies, the impact of EPO treatment on melanoma cell growth was further confirmed by *in vitro* proliferation experiments. Since cell growth is frequently accompanied by an increase in de novo protein synthesis, we measured uptake of AHA and found that EPO treatment of B16 cells caused a dose-dependent increase in protein synthesis. At the molecular level, and consistent with previous studies, we observed that EPO-induced proliferation of B16 cells was mediated by Jak2. However, contrary to observations in other tumor cells, Stat3 and Stat5 were not involved in EPO-signaling in B16 cells. Instead, our result indicate that treatment of B16 melanoma cells with EPO results in activation of several cell signaling pathways that eventually lead to phosphorylation of 4E-BP1. In addition to Jak2, the kinases Akt, and Erk were both phosphorylated within 30 minutes of EPO exposure. Since Erk, a kinase that plays a major role in mediating oncogenic signals, is hyperactivated in 90% of human melanomas [36], the additional stimulatory effects by EPO may critically affect survival and proliferation of melanoma cells. Importantly, our studies demonstrate, for what we believe to be the first time that stimulation of melanoma cells with therapeutic concentrations of EPO induced an increase in phosphorylation, and therefore activation of the translational regulatory protein eIF4E. Interestingly, experimental data indicate that eIF4E has a functional role not only in the regulation of protein synthesis but also in cellular transformation, tumor growth, and malignancy by selectively and disproportionately affecting the translation of mRNAs encoding key malignancy-related proteins including VEGF, c-myc, and cyclin D1 [37]. Transgenic mice overexpressing eiF4E develop lymphomas, angiosarcomas, lung carcinomas and hepatomas [38]. In addition, clinical data indicate that elevated total eIF4E levels along with increased phosphorylation of 4EBP1 is associated with disease progression and poor patient survival in breast, prostate and ovarian cancers.. Accordingly, targeting eIF4E or the eIF4F complex has become an attractive, novel anticancer strategy [39]. Experimental models show that the 4E-BPs are involved in tumorigenesis and that 4E-BP1 overexpression suppresses tumor formation and growth [40]. Indeed, Graff et al. [18] reported that intravenous eIF4E-specific antisense oligonucleotides administration in nude mice bearing PC-3 human prostate cancer or MDA-MB-231 human breast cancer xenografts, significantly suppressing tumor growth without eliciting toxicity, and also inhibited the formation of vessel-like structures by cultured human endothelial cells. Using eIF4E siRNAs, we observed that downregulation of eIF4E expression in B16 melanoma cells can profoundly reverse the EPO-induced increase in melanoma cell proliferation, suggesting that EPO-mediated melanoma tumor cell growth is eIF4E-dependent. Alterations in downstream targets of Akt such as the mammalian target of rapamycin (mTOR) pathway have recently also been shown to have significant effects on tumor progression in melanoma but we were unable to find any published literature supporting a relationship between EPO-mediated tumor proliferation and the mTOR pathway [41]. In summary, to the best of our knowledge the data presented herein is the first report linking EPO-mediated tumor proliferation to eIF4E activation. Since, treatment of various cancer cell lines with EPO *in vitro* elicits secretion of angiogenic growth factors and promotes cell proliferation [42], processes regulated by eIF4E, therapeutic suppression of translation initiation factor eIF4E in EPO-treated patients might provide an attractive new approach to the treatment of human malignancies.

## MATERIALS AND METHODS

### Cell culture, EPO stimulation and protein isolation

B16F10 (B16) melanoma cells were cultured in RPMI 1640 supplemented with 10% fetal bovine serum (FBS), 2 mM L-glutamine and penicillin/streptomycin at 37°C in 5% CO_2_, as described previously [43]. WT7 dry cell pellets were a gift from Dr. Monique Verhaegen (University of Michigan, Ann Arbor, MI). To test the effect of EPO treatment on B16 cells, the cells were plated in Petri dishes (0.5M/ml), serum-starved (1% FBS) for 24 hrs, then left unstimulated or stimulated with 10 U/ml EPO (Epogen, Amgen, Thousand Oaks, CA) for the indicated time at 37°C. Following treatment, the cells were washed with ice cold phosphate-buffer saline (PBS), lysed directly in radioimmunoprecipitation (RIPA) buffer containing 1X Halt protease inhibitor cocktail (Pierce, Rockford, IL). Insoluble material was removed by centrifugation at 16,000 g for 10 min and the supernatant saved as whole cell lysate. The amount of cellular protein present was measured using the BCA Protein Assay (Pierce).

### In *vivo* melanoma tumor model

Six-week-old female C57BL/6 (B6, H-2^b^) mice were purchased from Harlan Laboratory (Indianapolis, IN) and housed in a pathogen-free environment controlled for temperature and humidity. Procedures involving the animals and their care were conducted in accordance with the guidelines for animal treatment at the University of Michigan. The B16 cells (5 × 10^5^ cells per mouse) were injected subcutaneously into the right flank of C57BL/6 mice on day 0. When the tumors were palpable (around day 7), EPO (30U) was injected into mice subcutaneously three times a week. Controls were subcutaneously injected with phosphate-buffered saline (PBS). The mice were followed for a total of three weeks and tumor size was quantified every other day by measuring in a blinded fashion two perpendicular tumor diameters using a caliper, and recorded as the product of two orthogonal diameters (a × b). Hematocrit was determined in all animals by collecting 50 μl of blood in a capillary tube from the saphenous vein, then centrifuging for 1 min and calculating hematocrit from the ratio of the length of packed red blood cells (RBCs) to that of plasma as previously described [44].

### Western blotting

Twenty μg from each sample were diluted in Laemmli loading buffer, denatured by boiling for 5 minutes, subjected to sodium dodecyl sulfate-polyacrylamide gel electrophoresis and transferred to nitrocellulose membranes (Biorad, Hercules, CA). Membranes were blocked for 2 hrs in TBS containing 0.1% Tween 20 (Sigma-Aldrich) and 5% nonfat dry milk (Bio-Rad). After an overnight incubation at 4°C with the primary antibodies in TBS, 0.1% Tween 20, and 5% nonfat dry milk or bovine serum albumine (BSA), blots were washed three times with TBS containing 0.1% Tween (TBS-T) and incubated with a HRP-linked secondary antibodies for one hour. After three washes with TBS-T, the membranes were treated with ECL detection system (GE Healthcare, Piscataway, NJ), exposed to x-ray film (Denville Scientific, Metuchen, NJ) and developed to visualize the labeled protein bands. Molecular mass was estimated by comparison of sample bands with prestained molecular mass marker (Bio-Rad). For quantitative studies, the bands on x-ray films were scanned using a photodocumentation system (Alpha Innotech) and analyzed with ImageQuant 5.2 software (GE Healthcare). Where indicated, blots were stripped and reblotted with the corresponding antibody. Values were normalized respect to b-actin or total antibody as indicated.

### Antibodies

The following primary Abs were used: rabbit polyclonal anti-phospho-Jak2 (Tyr^1007/1008^), anti-phospho-Stat3 (Tyr^705^), anti-phospho-Stat5 (Tyr^694^), anti-phospho-Akt (Ser^473^), anti-phospho-eIF4E (Ser^209^), anti-phosp ho-4E-BP1 (Ser^65^) used at 1/1000 dilution (Cell Signaling Technology), and mouse monoclonal anti-β-actin (1/5000; Sigma-Aldrich; St. Louis, MO) and anti-GAPDH (1/5000; Abcam, Cambridge, MA) antibodies. Goat anti-mouse EPOR (use at 0.2ug/ml) was purchased from R&D, and anti-total 4E-BP1, anti-total Jak2, anti-total eIF4E, and anti-total Akt were from cell Signaling. Secondary Abs included: anti-goat IgG-HRP (1/2500; Santa Cruz, Santa Cruz, CA), anti-rabbit and anti-mouse IgG-HRP (1/2000; Cell Signaling Technology).

### Proliferation assays

The incorporation of 5-bromo-2′-deoxyuridine (BrdU) was used as a measurement of cell proliferation and assessed by enzyme linked immunosorbent assay (ELISA) with the BrdU Labeling and Detection Kit (Roche), according to the manufacturer's instructions. Briefly, B16 cells (10^4^ cells per well, in quadruplicate) were incubated with 10 μM BrdU for 12 hours after EPO treatment. In the respective background controls BrdU was omitted. Where indicated, anti-human EPO antibody (30μg/ml; R&D) or the Jak2 inhibitor Tyrphostin AG490 (50μM; Sigma Aldrich) were added one hour prior to EPO treatment. For controls, cells were left untreated or incubated alone with either anti-EPO antibody, Tyrphostin AG490, or Mitomycin C (5 μg/ml). Absorbance was measured in an ELISA plate reader at 370 nm (reference wavelength at 492 nm) and was directly correlated to the level of BrdU incorporation into cellular DNA.

### RNA quantification by multiplex real-time RT-PCR

Total RNA was isolated using the RNeasy Mini Kit (QIAGEN, Valencia, CA) and concentration was determined using a UV-Vis spectrophotometer NanoDrop ND-1000 (NanoDrop Technologies, Wilmington, USA). EPOR transcripts were amplified using a Rotor-Gene 3000 (Corbett Research) and detected with the Universal ProbeLibrary system (Roche Diagnostics, Boulder, CO). Briefly, a total of 200 ng of RNA was converted to cDNA and amplified in one step using Quanti-Tect SYBR Green RT-PCR kit (Qiagen) according to the manufacturer's instructions. The following conditions were used: reverse transcription at 50°C for 30 min, denaturation at 95°C for 15 min, amplification at 94°C for 6s, 55°C for 8 s, and 72°C for 12 s for a total of 40 cycles. Product quality was determined by melting curves. A series of five dilutions of one RNA sample were also included to generate a standard curve, and this was used to obtain relative concentrations of the transcript of interest in each of the RNA samples. In each experiment, water was included as a negative control to rule out primer dimer formation. EPOR specific primers and the most appropriate universal probe were designed using the Roche Universal ProbeLibrary Assay Design Center as follow: EPOR-probe 97, forward 5′–ggcaggagggacacaaag-3′ and reverse 5′-cgcaggttgctcagaacac-3′. All primers were produced by Integrated DNA Technologies, and all probes were from Roche Diagnostics. Universal ProbeLibrary Mouse GAPD Gene Assay (Roche Diagnostics) was used for further normalization.

### Metabolic labeling of newly synthesized proteins

New protein synthesis was measured using L-azidohomoalanine (AHA), an amino acid analog of methionine containing an azide moiety, and a Click chemistry approach from Invitrogen, according to the manufacturer's instructions. Briefly, cells were incubated in serum- and methionine-free RPMI for one hour followed by growth in 1% FBS- methionine-free medium supplemented with 50 μM mM AHA in the presence or absence of EPO (10 U/ml). AHA was omitted from some cultures as a negative control. After 2, 4 and 8 hrs, the cells were fixed with 4% paraformaldehyde in PBS for 15 min, and then permeabilized with 0.25% triton X-100 in PBS for 15 min. Incorporation of AHA was detected following a click reaction, the chemoselective ligation between an azide and an alkyne, with Alexa Fluor 488 alkyne. Fluorescence was then quantified on a FACSCalibur (Becton Dickinson, San Jose, CA).

### Transfection of small interfering RNA (siRNA)

B16 cells were plated overnight in 12-well plates (5 × 10^4^ cells/ cm^2^) in serum-containing media without antibiotics. Cells were then transfected with mouse eIF4E siRNA (100 nM; Cell Signaling) using DharmaFECT™ buffer 4 (1.4 μl/cm^2^ for each well; Dharmacon) according to the manufacturer's instructions. Seventy-two hours after transfection, cells were treated for BrdU incorporation or whole protein extracts were prepared. Non-targeting control GAPDH siRNA (Dharmacon) was used as control at corresponding concentration. Cell viability was assessed by detection of fluorescent labeled SignalSilence Control siRNA (Cell Signaling).

### Statistical analysis

Results are expressed as means ± SEM. Statistical analyses were performed using Student's *t-*test, and *p* ≤ 0.05 was taken as statistically significant. For multiple comparisons, 2 tailed student *t-*test was used.
